# DNA Barcoding Evaluation and Its Taxonomic Implications in the Species-Rich Genus *Primula* L. in China

**DOI:** 10.1371/journal.pone.0122903

**Published:** 2015-04-13

**Authors:** Hai-Fei Yan, Yun-Jiao Liu, Xiu-Feng Xie, Cai-Yun Zhang, Chi-Ming Hu, Gang Hao, Xue-Jun Ge

**Affiliations:** 1 Key Laboratory of Plant Resources Conservation and Sustainable Utilization, South China Botanical Garden, Chinese Academy of Sciences, Guangzhou, China; 2 College of Life Sciences, South China Agricultural University, Guangzhou, China; 3 Tropical Agriculture department, Guangdong Agriculture Industry Business Polytechnic College, Guangzhou, China; Chinese Academy of Medical Sciences, Peking Union Medical College, CHINA

## Abstract

The genus *Primula* is extremely diverse in the east Himalaya-Hengduan Mountains (HHM) in China as a result of rapid radiation. In order to overcome the difficulty of morphological classification of this genus, we surveyed three plastid regions (*rbc*L, *mat*K, and *trn*H*-psb*A) and two nuclear markers (ITS and ITS2) from 227 accessions representing 66 *Primula* species across 18 sections, to assess their discriminatory power as barcodes. We found that ITS alone or combined with plastid regions showed the best discrimination across different infrageneric ranks and at species level. We suggest *rbc*L *+ matK* + ITS as the first choice at present to barcode *Primula* plants. Although the present barcoding combination performed poorly in many closely related species of *Primula*, it still provided many new insights into current *Primula* taxonomy, such as the underlying presence of cryptic species, and several potential improper taxonomic treatments. DNA barcoding is one useful technique in the integrative taxonomy of the genus *Primula*, but it still requires further efforts to improve its effectiveness in some taxonomically challenging groups.

## Introduction

There is a critical need for rigorously delineated species for many theoretical studies and practical applications [1]. However, using traditional morphology-based taxonomy is difficult to discover morphologically cryptic taxa [2]. Species that are the product of rapid radiations within single genera can represent suites of morphologically similar taxa that are difficult to distinguish both in the field and the herbarium [3]. DNA barcoding is a valuable addition to the taxonomic tool box. After 10 years development of DNA barcoding, it has been found that large genera with rapid evolutionary radiations still pose a significant challenge for a universal barcoding system [4,5,6]. In order to understand better the overall discriminatory power of the plant barcoding loci, future work should focus on groups that experienced rapid evolutionary radiations, for example, the closely related species within a single genus.

*Primula* L. is an extraordinarily species-rich group within the east Himalaya-Hengduan Mountains (HHM) in China. The genus consists of about 500 species with over 300 of these found in China, and most of them (approximately 200 species) are restricted to populations in Southwest China, and are mainly confined to the HHM [7]. The HHM and its adjacent regions have been considered to represent the modern diversification centre of the genus [8]. The exceptionally high *Primula* species and/or lineage diversity in China occurred no more than 10 Mya [9], and may have been triggered by the extensive uplifts of the Qinghai-Tibet Plateau (QTP) since the early Miocene and strengthened by topographical complexity of the QTP and climate oscillations during the Quaternary [10]. Like other large plant groups co-occurring on the QTP (such as *Pedicularis*, *Rhododendron*, *Gentiana* and *Saussurea*), *Primula* is a taxonomically challenging group because: 1) many key diagnostic features are tiny and empirical, and cannot be determined correctly by non-specialists, these features include the shape of calyx and bracts [7]; 2) many dwarf species (such as *Primula* section *Minutissimae*) are too small in size to separate; and 3) frequent hybridization or introgression can confuse the *Primula* species boundaries. *Primula* species, even distantly related ones, can be hybridized readily in greenhouse conditions [11] and in the wild, as reported recently [12–14]. In addition, new *Primula* species in the HHM and adjacent area have been described a number of times in recent years [15–20]. This suggests that the species diversity of *Primula* is still underestimated. Although monographs describing *Primula* do exist [7,11,21,22], the use of keys for the genus requires a high level of specialized expertise. A more efficient approach to facilitate delimitating *Primula* species and discovering cryptic species or lineages in the genus is urgently required. Despite the promise of DNA barcoding, only a few studies have used it in plant groups that have a high diversity in the HHM or in neighboring regions [23–27].

Although the limited ability of DNA barcoding to discriminate species in large genera is well known, the following questions are still unclear: 1) to what extent could DNA barcoding discriminate infrageneric levels (i.e., subgenus, section, and series) within large genera? 2) Could DNA barcodes discriminate between certain closely related species pairs? 3) In rapidly evolved genera, could DNA barcoding reveal cryptic species? As a typical rapidly evolved plant taxon in the HHM, the genus *Primula* provides a good opportunity to answer these questions. In the current study, we sampled 66 species representing 18 sections of *Primula* in China; these contained many closely related groups. The discriminatory ability of three common plastid barcoding candidates (*rbc*L, *mat*K, and *trn*H*-psb*A) and nuclear regions (ITS and ITS2) were evaluated.

## Materials and Methods

### Ethics statement

All samples employed in this study are not endangered nor protected in the sampled area, and none of the sampled locations are privately owned or protected by any law. No specific permits were required for the described field studies.

### Taxon sampling, DNA extraction and sequencing

During this study we examined a total of 227 accessions of 66 *Primula* species from 18 of the 24 sections of the genus in China recognized by Hu [21]. We used *Omphalogramma delavayi* Franch. as an outgroup [28,29]. In order to explore the pattern of genetic variability in morphological species, more than two individuals of each species were collected. Taking account of the effect of geographical sampling scale on DNA barcoding [30], more individuals (> 10) were sampled from widespread species, such as *P*. *secundiflora* Franch. and *P*. *Poissonii* Franch., across their ranges to allow for their intraspecific variability.

To test the effectiveness of DNA barcoding in more closely related groups, section *Proliferae* was exhaustively sampled in this study. There are approximately 23 species in this section [11]. In China, nineteen species have been described [7,8,21], and a new record species, *P*. *burmanica* Balf. f. et Ward, in the section was recently discovered on the south side of Ailao Mountain in Simao (Szemao) region, China (Yan *et al*., unpublished data). We collected 84 accessions representing all species of the section in China except *P*. *stenodonta* Balf. f. ex W. W. Smith et Fletcher. In addition, we selected several of the most closely related species groups in the genus, such as *P*. *chungensis* Balf. f. et Ward vs. *P*. *cockburinana* Hemsl., *P*. *ovalifolia* Franch. vs. *P*. *tardiflora* C. M. Hu, *P*. *prattii* Hemsl. vs. *P*. *pulchella* Franch., *P*. *fasciculata* Balf. f. et Ward vs. *P*. *munroi* ssp. *yagongensis* (Petitm) W. W. Smith et Forr., and the *P*. *poissonii* complex. Collection details, voucher numbers, taxonomy, and GenBank accession numbers are listed in S1 Table.

Genomic DNA was extracted from silica gel-dried leaf material following a modified version of the cetyltrimethyl ammonium bromide (CTAB) protocol of Doyle & Doyle [31]. Five candidate DNA barcodes, containing two coding plastid genes (*rbc*L and *mat*K), one intergenic plastid spacer (*trn*H*-psb*A), the nuclear ribosomal internal transcribed spacer (ITS, including ITS1, 5.8s and ITS2) and the internal transcribed spacer2 (ITS2), were evaluated in this study. *Rbc*L was amplified using the primer combination (*rbc*La_f and 724R) as suggested by Fay *et al*. [32] and Kress & Erickson [33], respectively. The amplification of *mat*K was achieved using the primer pair 3F-KIM and XF ([34]; Kim unpublished data). For *trn*H*-psb*A, the primers trnH05 and psbA3 were used [35,36]. ITS was amplified with the primers proposed by White *et al*.[37]. PCR amplification and sequencing conditions followed Yan *et al*. [24]. ITS2 was retrieved from the ITS data in this study.

### Data analyses

Sequences for each marker were aligned with Muscle 3.8 [38] and then manually adjusted using Se-Al 2.0a11 [39]. We focused on evaluating five single markers and their combinations (*rbc*L *+ mat*K, *rbc*L *+ mat*K *+ trn*H*-psbA*, *rbc*L *+ mat*K *+* ITS, *rbc*L *+ mat*K *+* ITS2, *rbc*L *+ mat*K *+ trn*H*-psb*A + ITS, and *rbc*L *+ mat*K *+ trn*H*-psb*A + ITS2). For the pair-wise genetic distance (PWG-distance) method, the genetic pairwise distance was determined by MEGA6 using the Kimura two-parameter distance model (K2P) with pairwise deletion of missing sites [40]. Three parameters (average intraspecific distance, average theta (ө), and coalescent depth) were calculated for all markers. In order to evaluate the ‘local’ barcoding gap for each species [41,42], we plotted the maximum intraspecific divergences against the smallest interspecific distances for each species [41,43].

To test whether accurate species assignments can be made among the samples using a single marker or combinations of markers, we used another two distance-based methods the ‘best match’ (BM) and ‘best close match’ (BCM) using the TaxonDNA/Species Identifier 1.7.7-dev3 program [44]. BM assigns the query to the species with the smallest distance sequence, whereas BCM only identifies the query when the closest sequence is within a distance threshold. The threshold value is determined by using the distance less than 95% of all intraspecific distances, which was calculated by the pairwise summary function [44].

For the tree-building method, we calculated the proportion of monophyletic groups using a Neighbor-Joining (NJ) tree. The test was performed using PAUP* v4b10 with the K2P model [45]. If all individuals of a species cluster together with a bootstrap value above 70%, then the species was considered as having been successfully identified.

## Results

### Sequence characteristics and genetic divergence

**Fig 1:**
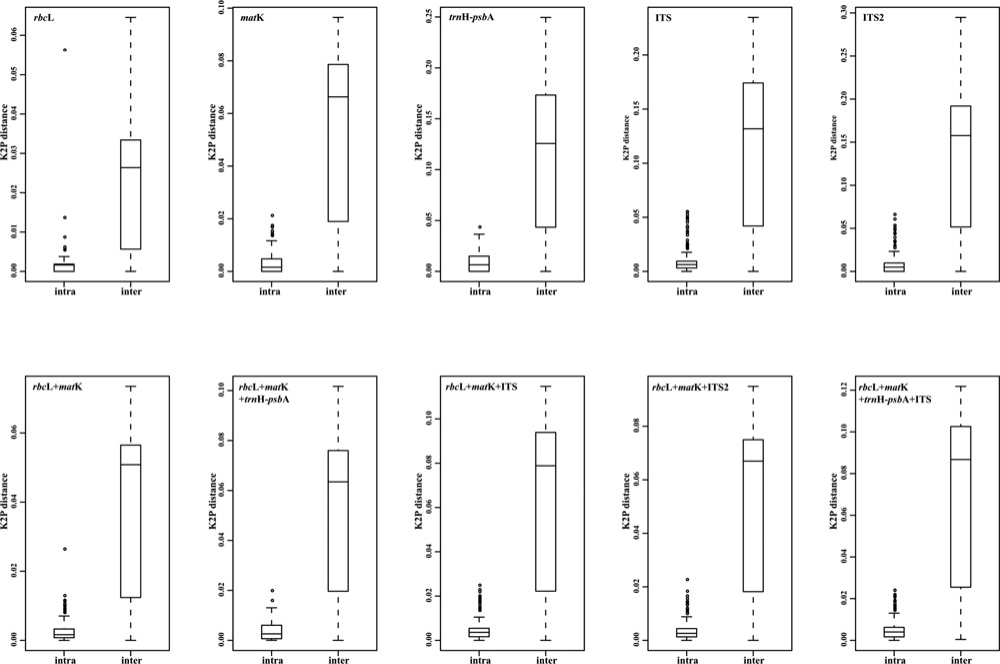
Comparisons of the distribution ranges of inter- and intraspecific distances using boxplots.

All plastid markers (*rbc*L, *mat*K, *trn*H*-psb*A) were successfully amplified across all individuals, but amplification of ITS failed in two *Primula* species (*P*. *virginis* Lévl. and *P*. *duclouxii* Petitm.) and one accession of *P*. *gemmifera* Batal. (GXJ253, voucher: Hao940) in this study (S1 Table). The characteristics of the five DNA markers are presented in Table 1. Overall, the aligned length of the five markers ranged from 241 bp (ITS2) to 857 bp (*trnH-psbA*). The proportion of variable sites were the lowest for *rbc*L and highest for ITS2. *Rbc*L exhibited the lowest intra-specific and/or inter-specific divergence as well, whilst *trn*H*-psb*A showed the highest intra-specific divergence (0.87%), followed by ITS2 (0.80%). However, the greatest interspecific distance was found in ITS2 (12.73%), followed by *trnH-psbA* (11.69%). The box-and-whisker plots (Fig 1) indicate the distance distribution of inter- and intra-specific distances for all single markers.

**Table 1:** Summary of genetic variability and sequence characteristics of the candidate barcodes and their main combinations in this study.

	*rbc*L	*mat*K	*trn*H*-psb*A	ITS	ITS2	R + M	R + M + T	R + M + I	R + M + I2	R + M + T + I
Aligned length (bp)	614	718	857	680	241	1333	2191	2015	1575	2872
Average intra-distance	0.14%	0.33%	0.87%	0.75%	0.80%	0.24%	0.36%	0.41%	0.32%	0.47%
Average inter-distance	2.14%	5.12%	11.69%	11.10%	12.73%	3.71%	5.06%	6.04%	4.92%	6.70%
Average theta (ө)	0.17%	0.24%	0.25%	0.48%	0.58%	0.21%	0.21%	0.29%	0.26%	0.29%
Coalescent Depth	5.57%	2.04%	4.15%	5.30%	6.36%	2.58%	1.91%	2.39%	2.19%	2.31%
Proportion of variable sites	15.79%	33.43%	47.37%	50.88%	51.19%	25.36%	32.63%	32.90%	29.21%	37.05%
Proportion of parsimony sites	12.38%	27.72%	32.56%	43.09%	48.13%	20.63%	25.33%	27.84%	24.57%	29.18%
Rate of PCR and sequencing success	100%	100%	100%	97.80%	97.80%	N/a	N/a	N/a	N/a	N/a

R, *rbc*L; M, *mat*K; T, *trn*H*-psb*A; I, ITS; I2, ITS2.

The mean intra and inter-specific genetic divergence for the main combinations varied in the ranges 0.24% to 0.47% and 3.71% to 6.70%, respectively (Table 1). The combination of *rbc*L *+ mat*K *+ trn*H-*psb*A + ITS exhibited the highest mean intra- and inter-specific distance, followed by *rbc*L *+ mat*K + ITS. The core barcode *rbc*L *+ mat*K exhibited the smallest intra-and inter-specific genetic difference (Table 1).

### Discrimination success of candidate barcodes

**Fig 2:**
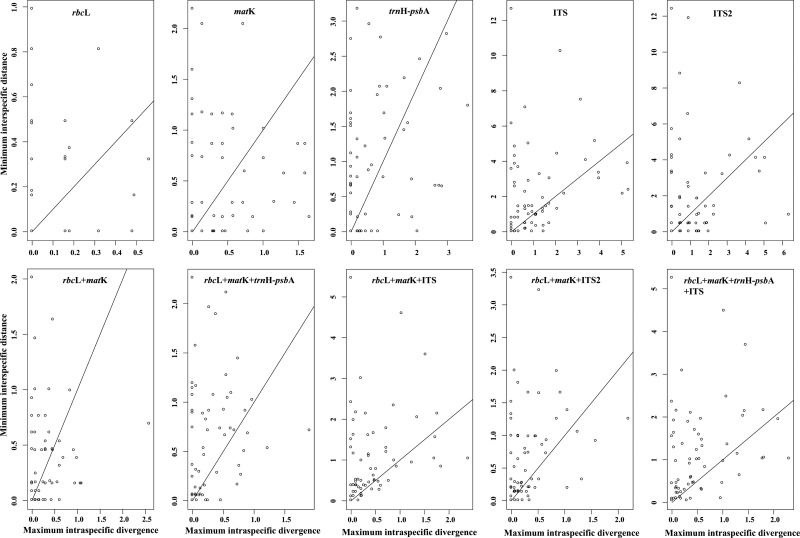
Relationships between inter- and intraspecific distance indicating the local gaps for species.

**Fig 3:**
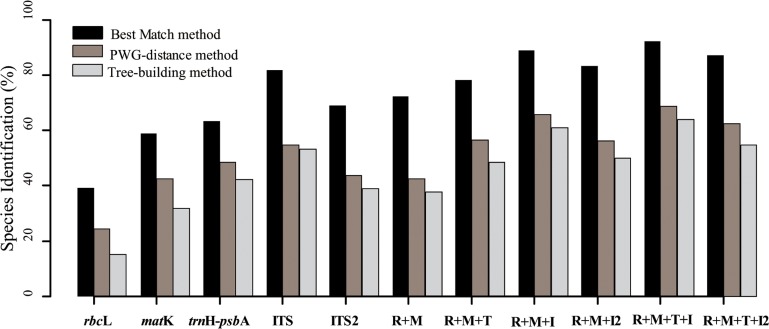
Species discrimination rates of several main barcodes in *Primula*. R, *rbc*L; M, *mat*K; T, *trn*H-*psb*A; I, ITS; I2, ITS2.

The local barcoding gap, with an interspecific distance larger than the intraspecific distance for a species, directly reveals the species discrimination ability of barcodes. The proportion of the local barcoding gap varied between the regions tested (Figs 2 and 3, S2 Table). ITS showed the best discriminatory power (54.69%) among the five single candidate barcodes, followed by *trn*H*-psb*A (48.40%). In contrast, *rbc*L provided the lowest discrimination rate (24.24%). Of all the combinations tested, the proportion of the barcoding gap of the core barcode combination (*rbc*L *+ mat*K) was the lowest (42.42%) (Fig 3, S2 Table), while *rbc*L *+ mat*K *+ trn*H*-psb*A + ITS exhibited the highest local barcoding gap (68.75%) followed by *rbc*L *+ mat*K + ITS and *rbc*L *+ trn*H*-psb*A + ITS (65.63%). *Trn*H*-psb*A and ITS2 individually and/or combined with other plastid markers did not perform well enough to discriminate *Primula* species in this study (Fig 3, S2 Table). For example, *rbc*L *+ mat*K *+ trn*H*-psb*A and *rbc*L *+ mat*K + ITS2 could identify 36 *Primula* species (56.65%), while *rbc*L *+ mat*K + ITS performed better and identified 65.63% of the *Primula* species.

Compared with the PWG-distance method, the BM and BCM analyses all showed better discrimination success. BCM always had a lower identification rate than BM analysis (S2 Table). Based on the BM model, ITS performed best among the five single DNA regions, and successfully assigned 81.98% sequences to the correct species (Fig 3). The identification rate of the two-locus combinations ranged from 71.36% to 89.63%. Among them, the core barcode combination *rbc*L *+ mat*K correctly identified 72.24% of specimens, which was only slightly better than *rbc*L *+ trn*H*-psb*A (71.36%). For three-locus combinations, *mat*K *+ trn*H*-psb*A + ITS, *rbc*L + *trn*H*-psb*A + ITS, and *rbc*L *+ mat*K + ITS provided similar discrimination rates (90.99%, 90.54%, and 89.18%), followed by *rbc*L *+ mat*K *+ trn*H-*psb*A (78.41%). In addition, combinations with ITS2 always produced a lower identification rate compared to combinations with ITS (Fig 3, S2 Table).

The tree-building method provided a similar result to the distance-based method. In this analysis, we found that ITS was the best of all single markers, successfully identifying 53.13% of species. Of the combinations, *rbc*L *+ mat*K showed the poorest discriminatory power (37.88%), while *rbc*L *+ mat*K *+ trn*H*-psb*A + ITS was the best one with a 64.06% discrimination rate, followed by *rbc*L *+ mat*K + ITS and *mat*K *+ trn*H*-psb*A + ITS (60.94%) (Fig 3, S2 Table).

**Fig 4:**
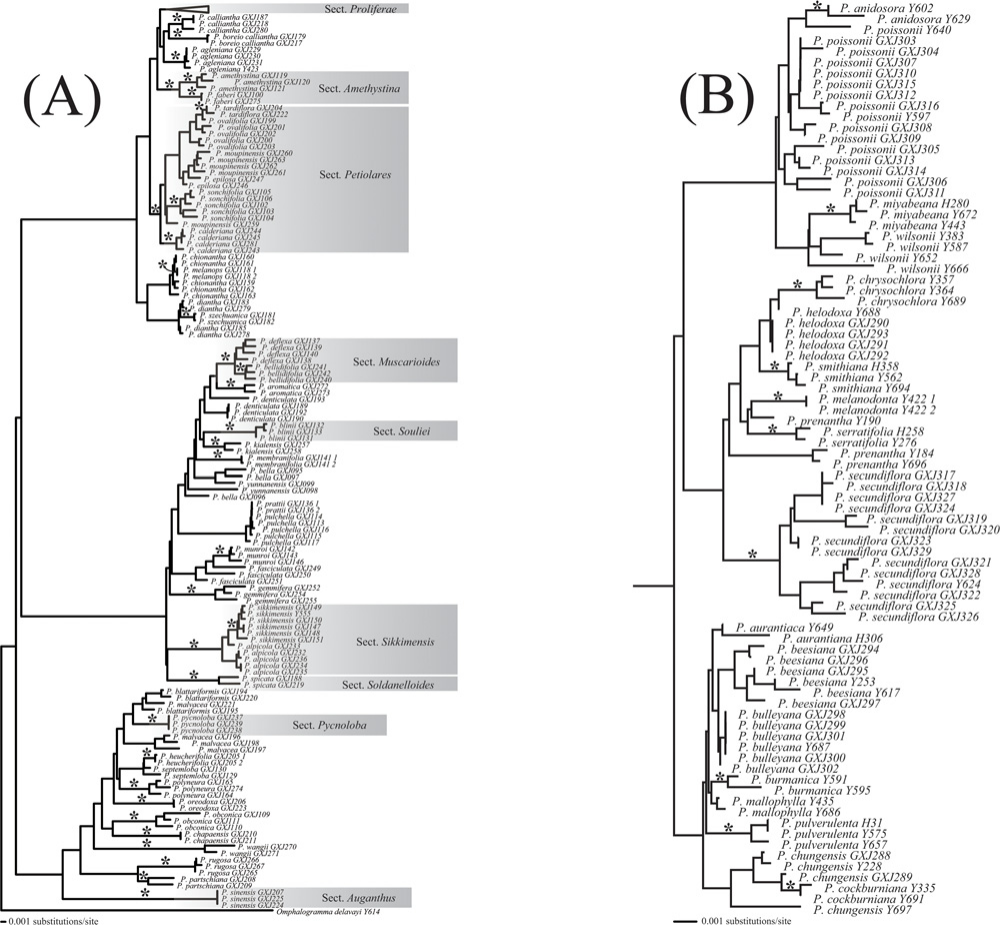
Neighbor-joining tree based on the combination *rbc*L *+ matK* + ITS with the K2P distance model. (A) The whole tree of *Primula* except section *Proliferae*. (B) The tree of section *Proliferae*. Asterisks along branches indicate monophyletic species with bootstrap values above 70%. Accessions are suffixed by sample ID. Monophyletic sections are highlighted with grey shading.

When we considered the previously recognized infrageneric taxa (the twenty four sections, [21]), *rbc*L and *trn*H-*psb*A each only identified four sections (S1 Fig). The discrimination rate of ITS was the best among all single barcodes, distinguishing eight sections (section *Pycnoloba*, section *Auganthus*, section *Souliei*, section *Soldanelloides*, section *Sikkimensis*, section *Amethystina*, section *Muscarioides*, and section *Petiolares*) (S1 Fig). Among the main combinations, the core barcode (*rbc*L *+ mat*K) only successfully identified five sections (section *Pycnoloba*, section *Auganthus*, section *Souliei*, section *Soldanelloides*, and section *Sikkimensis*), followed by the combinations *rbc*L *+ mat*K *+ trn*H*-psb*A + ITS2, *rbc*L *+ mat*K *+ trn*H*-psb*A + ITS and *rbc*L *+ mat*K *+ trn*H*-psb*A, which all identified the same eight same sections as ITS. In contrast, *rbc*L *+ mat*K + ITS was the best combination, and was able to discriminate nine sections (including section *Proliferae*) (Fig 4). Our sampling represented four subgenera (subgenus *Auriculastrum*, subgenus *Auganthus*, subgenus *Carolinella* and subgenus *Aleuritia*) according to the revised classification of *Primula* [11], nevertheless, the majority of DNA barcodes singly or jointly could only separate out subgenus *Auriculastrum* correctly.

### Discrimination ability of DNA barcoding in closely related groups

Section *Proliferae* is an example containing closely related taxa that is suitable for testing the discriminatory performance of DNA barcoding. Using the tree-building method, the core barcode (*rbc*L *+ mat*K) could only correctly identify *P*. *smithiana* Craib with a relatively high bootstrap value (i.e. over 70%), whereas ITS alone could distinguish five species (S1 Fig). *rbc*L *+ mat*K + ITS was the most efficient and precise combination in this study, as stated above, but it only discriminated 10 species correctly (52.63%) in this section (Fig 4). Section *Proliferae* contained three taxonomically challenging groups (or species complexes). Although the three groups could be easily distinguished by *rbc*L *+ mat*K + ITS, the species within each group were difficult to discriminate using the current barcodes singly and/or in combination. For example, the *P*. *poissonii* complex is resolved as monophyletic with high support by *rbc*L *+ mat*K + ITS, however, only two narrowly distributed species (*P*. *anisodora* Balf. f. et. Forr. and *P*. *miyabeana* Ito et Kawakami) could be readily distinguished (Fig 4).

Compared with section *Proliferae*, the discrimination performance of DNA barcoding in other *Primula* species was much better (64.44%, based on the tree-building result of *rbc*L *+ mat*K + ITS). However, we found that a failure often occurred in the most closely related species groups (Table 2), such as *P*. *chungensis* vs. *P*. *cockburinana*, *P*. *ovalifolia* vs. *P*. *tardiflora*, *P*. *prattii* vs. *P*. *pulchella*, and *P*. *fasciculata*vs. *P*. *munroi* ssp. *yagongensis*. In addition, some species showed extremely high intraspecific divergence (>1%); these included *P*. *moupingensis* Franch., *P*. *bella* Franch., *P*. *fasciculata*, *P*. *malvacea* Franch. and *P*. *yunnanensis* Franch. (Table 2). Most of the species with extremely high intraspecific divergence cannot be correctly distinguished by any of the three methods, which probably indicates their status should be further examined.

**Table 2:** Summary of the candidate barcode *rbc*L *+ mat*K + ITS divergence pattern for unidentified species.

Taxon	The nearest relative	Mean intraspecific divergence (%)	Maximum intraspecific distance (%)	Minimum interspecific distance (%)
*P*. *prattii*	*P pulchella*	0	0	0
*P*. *pulchella*	*P*. *prattii*	0.1	0.15	0
*P*. *burmanica*	*P*. *mallophylla*	0.19	0.21	0.16
*P*. *chungensis*	*P*. *cockburniana*	0.17	0.21	0.11
*P*. *bulleyana*	*P*. *aurantiaca*	0.32	0.21	0.11
*P*. *aurantiaca*	*P*. *bulleyana*	0.13	0.22	0.11
*P*. *chrysochlora*	*P*. *helodoxa*	0.13	0.26	0.26
*P*. *poissonii*	*P*. *anidosora*	0.12	0.38	0.38
*P*. *chionantha*	*P*. *melanops*	0.18	0.41	0.1
*P*. *beesiana*	*P*. *bulleyana*	0.02	0.43	0.21
*P*. *wilsonii*	*P*. *miyabeana*	0.25	0.48	0.37
*P*. *septemloba*	*P*. *heucherifolia*	0.32	0.56	0.46
*P*. *prenantha*	*P*. *helodoxa*	0.21	0.58	0.26
*P*. *ovalifolia*	*P*. *tardiflora*	0.42	0.61	0.36
*P*. *alpicola*	*P*. *sikkimensis*	0.26	0.77	0.48
*P*. *blattariformis*	*P*. *malvacea*	0.6	0.93	0.83
*P*. *moupinensis*	*P*. *epilosa*	0.54	1.24	0.61
*P*. *denticulata*	*P*. *kialensis*	0.48	1.6	1.3
*P*. *bella*	*P*. *yunnanensis*	0.9	1.7	1.03
*P*. *fasciculata*	*P*. *munroi*	0.99	1.72	1.55
*P*. *malvacea*	*P*. *blattariformis*	0.85	1.84	0.83
*P*. *yunnanensis*	*P*. *bella*	1.82	2.39	1.03

## Discussion

### The resolution of the tested DNA markers in *Primula*

In this study, all the three plastid regions tested individually showed a relatively low discriminatory efficacy ranging from 15.16% to 31.82% (based on monophyletic analysis) in *Primula* species (S2 Table). The core barcode *rbc*L *+ mat*K also provided low discrimination at a rate of 37.88% (S2 Table). One of the most promising supplementary plastid barcodes, *trn*H*-psb*A, varied in size from 154 bp (*P*. *poissonii*) to 523 bp (*P*. *polynuera* Franch.), so there were a large number of gaps in the alignment matrix. Based on tree-building analysis, *trn*H*-psb*A identified 42.19% of species; this was the best among the plastid regions but lower than the nuclear markers (ITS) (S2 Table). The combination of *trn*H*-psb*A with *rbc*L or *mat*K did not result in higher resolution (S2 Table), which demonstrated that *trn*H*-psb*A is not a preferred barcode in *Primula*.

The strong identification ability of ITS has been verified based on a comprehensive study [46], even in some complex plant groups, such as *Panassia* [25], *Ficus* [47], *Lysimachia* [27], and *Sisyrinchium* [48]. In this study, ITS exhibited the highest discriminatory power among all five markers, and any combinations with ITS were able to discriminate more species than combinations without ITS (Fig 3, S2 Table). Of the three-locus combinations, *rbc*L *+ mat*K + ITS and *mat*K *+ trn*H*-psb*A + ITS all distinguished 60.94% of monophyletic species, which was the best discrimination performance (Appendix S2). Therefore, as suggested by Yan *et al*. [24], *rbc*L *+ mat*K + ITS should be the first choice to barcode *Primula* plants. Compared with primer problems associated with ITS, ITS2 has conserved regions for designing universal primers, and can be readily amplified in various groups [49]. However, ITS2 itself or combined with plastid markers did not produce better results than ITS and/or corresponding combinations (S2 Table). We suggest that ITS2 may be an ideal supplementary barcode when ITS amplification fails.

### Discrimination performance on section rank in *Primula*

DNA barcoding should be able to help identify some groups within large genera, thus reducing the time required for morphological studies to produce definitive species lists. Although it is well known that DNA barcoding has difficulties in resolving closely related species, it is not clear whether such barcoding could identify samples correctly to section level within large genera. There are more than 200 *Primula* species concentrated in the HHM region in China [11]. *Primula* has always been divided into subgroups, usually with the rank of section [22,50]. In a well-accepted infrageneric system, Smith and Fletcher divided the genus into a total of 31 sections [22]. Twenty-four sections of the Chinese *Primula* were adopted by Hu [21].

In this study, DNA barcoding performed well for distinguishing sections, and could resolve nine of the current 18 sections [21]. However, of the resolved sections, three (namely section *Auganthus*, section *Souliei*, and section *Soldanelloides*) together with the monotypic section *Pycnoloba* were each represented by one species in the current study. Considering the fact that the phylogeny of many sections and their close relatives, such as section *Soldanelloides*, *Minutissimae*, and *Souliei*, still lack detailed studies [28,29], the discrimination rate of DNA barcoding would probably drop further if we expanded the number of members in these sections. These results demonstrated that DNA barcoding is useful in some sections of *Primula*. In addition to barcoding discriminatory ability, the infragenetric classification system will also influence the results. A reliable and well-recognized infrageneric rank in a large genus is a prerequisite for applying DNA barcoding. Therefore, a number of new sectional delimitations will be necessary in the genus *Primula* [11,28].

### Resolving ability of DNA barcoding in section *Proliferae*

The *Primula* section *Proliferae* is a well-delimited and natural group characterized by numerous whorls of flowers resembling candelabra [11]. It is mainly concentrated in the HHM [7]. In China, 19 species have been described and, with the exception of *P*. *miyabeana* (endemic to Taiwan), they are narrowly distributed in southwest China [7]. This section contains several taxonomically challenging groups, such as the *P*. *poissonii* complex, which consists of *P*. *anisodora*, *P*. *wilsonii* Dunn and *P*. *poissonii*, and the *P*. *beesiana* group with *P*. *beesiana* Forr., *P*. *bulleyana* Forr., *P*. *burmanica* and *P*. *pulverulenta* Duthie. This complex section provides a good example to test the discriminatory ability of candidate barcodes in closely related species, especially those formed through rapid evolutionary radiation.

Although the discriminatory power of DNA barcoding is limited in section *Proliferae* (discrimination rate of 52.63%), in the current study it confirmed the monophyly of section *Proliferae* (tree-building method) (Fig 4), and divided the section into three clades with high support (over 85%), which agree well with the study based on their morphology [51]. It is convenient for us to assign unknown *Primula* specimens to a rough position in the section. This could help to narrow the scope of identification. Within each complex or clade, DNA barcoding could still provide some clues for identification and taxonomic treatment. For example, *P*. *poissonii* and *P*. *anisodora* have the closest relationship and they were confirmed by the current barcodes (Fig 4), but only *P*. *anisodora* exhibited monophyly. DNA barcoding could also help to solve several classification disputes in this section. For example, barcoding supported treating *P*. *wilsonii* and *P*. *anisodora*, *P*. *burmarica* and *P*. *beesiana* as separate species [7,11,21,51] (Fig 4). Therefore, even for very closely related species, DNA barcoding may still provide help to some extent, and narrow the identification range.

It is well known that using the universal DNA barcode (two core barcodes and two alternative barcodes, *trn* H *-psb*A, ITS) it is almost impossible to separate very closely related species formed through rapid radiation. Therefore, species-specific barcodes need to be developed for difficult taxa [6]. These markers may be based on other rapidly evolved molecular markers such as low or single copy nuclear genes (e.g. *waxy* and *leafy*) [52] or even using high-throughput sequencing methods (such as RAD and GBS).

### Biological implications of DNA barcoding in *Primula*

Traditional taxonomy mainly depends on morphological diagnosis, and it should be corroborated by other sources of data, such as geographical, ecological, reproductive and DNA sequence information [53]. However, constructing a robust taxonomy for recently diverged plant taxa is more difficult, because they often show little difference in their morphological and genetic profiles. In addition, many other aspects could also cause the failure of DNA barcoding, such as imperfect taxonomy, interspecific hybridization, paralogy, and incomplete lineage sorting [42,52,54,55]. For many such taxa, DNA barcoding provides an opportunity to solve some of the taxonomic problems through discovering the underlying biological issues.

By surveying the non-monophyletic taxa at species level and examining genetic distance (Fig 4, Table 2), we filtered out barcoding failures in several species probably caused by incomplete lineage sorting. For example, narrowly distributed *P*. *tardiflora*, *P*. *prattii*, and *P*. *cockburiana* each experienced peripheral isolated speciation from their widely distributed relatives (putative parents) (*P*. *ovalifolia*, *P*. *pulchella*, and *P*. *chungensis*) [55]. The barcoding results were partially supported by a complementary phylogeographic study [56]. It is a question for taxonomy to reflect on these incomplete speciation processes by synonymizing the nested and parent species or elevating lineages in the paraphyletic lineage to species status [55]. In the context, we prefer to treat the nested and parent species as one species because of their similarity in morphology [7,21], but of course additional research is necessary.

Imperfect taxonomy in several plant and animal taxa has been detected by DNA barcoding (e.g. [27,42,57–59]), providing significant support for the taxonomic value of the technique. *P*. *bella* examined in this study is an excellent example of over-lumping in traditional taxonomy, as the species appeared polyphyletic and exhibited unexpectedly large intraspecific divergence (Table 2, Fig 4). Given the variable morphological characters (such as shape of bracts and the stem length), there are classification disputes about the delimitation of *P*. *bella* [7,11,21,60]. DNA barcoding supported the suggestion that the anomalous individual *P*. *bella* GXJ096 (voucher: Hao & Yan 956) should be raised to species status (*P*. *cyclostegia* Hand.-Mazz.) on the basis of its genetic profile, although additional work is essential to validate this as a robust species. A similar situation is also probably the case for *P*. *denticulata* Smith.

Discovering the potential presence of cryptic species and/or lineages is an important application of DNA barcoding, and this remains within the domain of taxonomy [53]. The taxonomic usefulness of DNA barcoding has been validated in a wide range of animals (see, for example [61–67]), but there are few studies of large plant groups that have recently experienced evolutionary radiation. It is plausible that the frequent occurrence of cryptic species in Chinese *Primula* represents adaptation to the variable habitats on the HHM and rapid radiation evolution in a relatively short time [7,9,11,21]. By iteratively reexamining peculiar specimens detected by DNA barcoding (such as *P*. *yunnanensis* GXJ099, *P*. *fasciculata* GXJ249, and *P*. *moupinensis* GXJ259) (Table 2, Fig 4), several tiny morphological or geographical divergences may be identified in these taxa, which indicate the possibility of cryptic species; however, further taxonomic scrutiny is required.

Another great challenge for barcoding plant species is linked to hybridization events [23,52,54,68]. Natural or artificial hybrids in *Primula* have been reported recently [11–14], and these may cause a failure in barcoding *Primula* species. In the current study, underlying hybridization might occur in *P*. *anisodora* and its most close relative *P*. *poissonii*. They were found in the same populations, and a putative hybrid (*P*. *poissonii* Y640) was also discovered (S2 Fig). Additional research is needed to resolve the biological situation (e.g. [69,70]).

## Conclusion

*Primula* species examined in the present study are difficult to distinguish using the core barcode (*rbc*L *+ mat*K). Another plastid marker, *trn*H*-psb*A, varied in size and exhibited lower discrimination compared to ITS, suggesting that it is not a suitable barcode for studies of *Primula*. In contrast, ITS showed the best discriminatory ability of all the single markers tested, discriminating 65.63% and 60.94% of species (according to the PWG-distance method and tree-building method) when combined with *rbc*L *+ mat*K, which performed best among all three-locus combinations. We propose that *rbc*L *+ mat*K *+* ITS should be treated as the first local barcode in the genus *Primula* at present, although its discrimination rates with respect to infrageneric rank and separating closely related *Primula* species are limited.

Despite the limited discrimination for closely related pairs, DNA barcoding provided many new insights into the current *Primula* taxonomy, such as detecting potential cryptic species, and revealing several probably improper taxonomic treatments. Obviously, it is difficult to resolve all closely related groups based on the current limited and relatively conserved molecular markers, especially in taxa such as *Primula*, which have experienced recent rapid radiation. Other more rapidly evolved molecular markers should be incorporated into future DNA barcoding projects, for example low or single copy nuclear genes, nuclear SNPs, nuclear SSRs [23,52], and the complete chloroplast genome [71–74]. As proposed by Twyford [6], we are building a robust phylogeny framework for the *Primula* section *Proliferae* using RAD (restriction-site-associated DNA, [75]), and expect to resolve the true evolutionary relationships; these may be necessary to develop robust species-specific barcodes in the future [6]. Overall, DNA barcoding is a useful technique for the integrative taxonomy of the genus, but it still requires further work to improve its value for studying taxonomically challenging groups.

## Supporting Information

S1 Fig Neighbor-joining trees based on candidate barcodes and their main combinations with K2P distance model. Asterisks along branches indicate monophyletic species with bootstrap values above 70%. Accessions are suffixed by sample ID. Monophyletic sections are highlighted with grey shading.
(PDF)

S2 Fig Three individuals of *Primula poissonii* complex and their flowers. (DOCX)

S1 Table Taxon, voucher, collection information, and Genbank accession numbers. (XLSX)

S2 Table Discrimination success based on different analysis methods. (DOCX)
