# Supplementary material for: DNA Barcoding Evaluation and Its Taxonomic Implications in the Species-Rich Genus Primula L. in China
Source: PLoS One. 2015 Apr 13;10(4):e0122903. doi: 10.1371/journal.pone.0122903 (PMC4395239; doi:10.1371/journal.pone.0122903)
Supplement: S1 Fig — Asterisks along branches indicate monophyletic species with bootstrap values above 70%. Accessions are suffixed by sample ID. Monophyletic sections are highlighted with grey shading. (PDF) [file pone.0122903.s001.pdf]

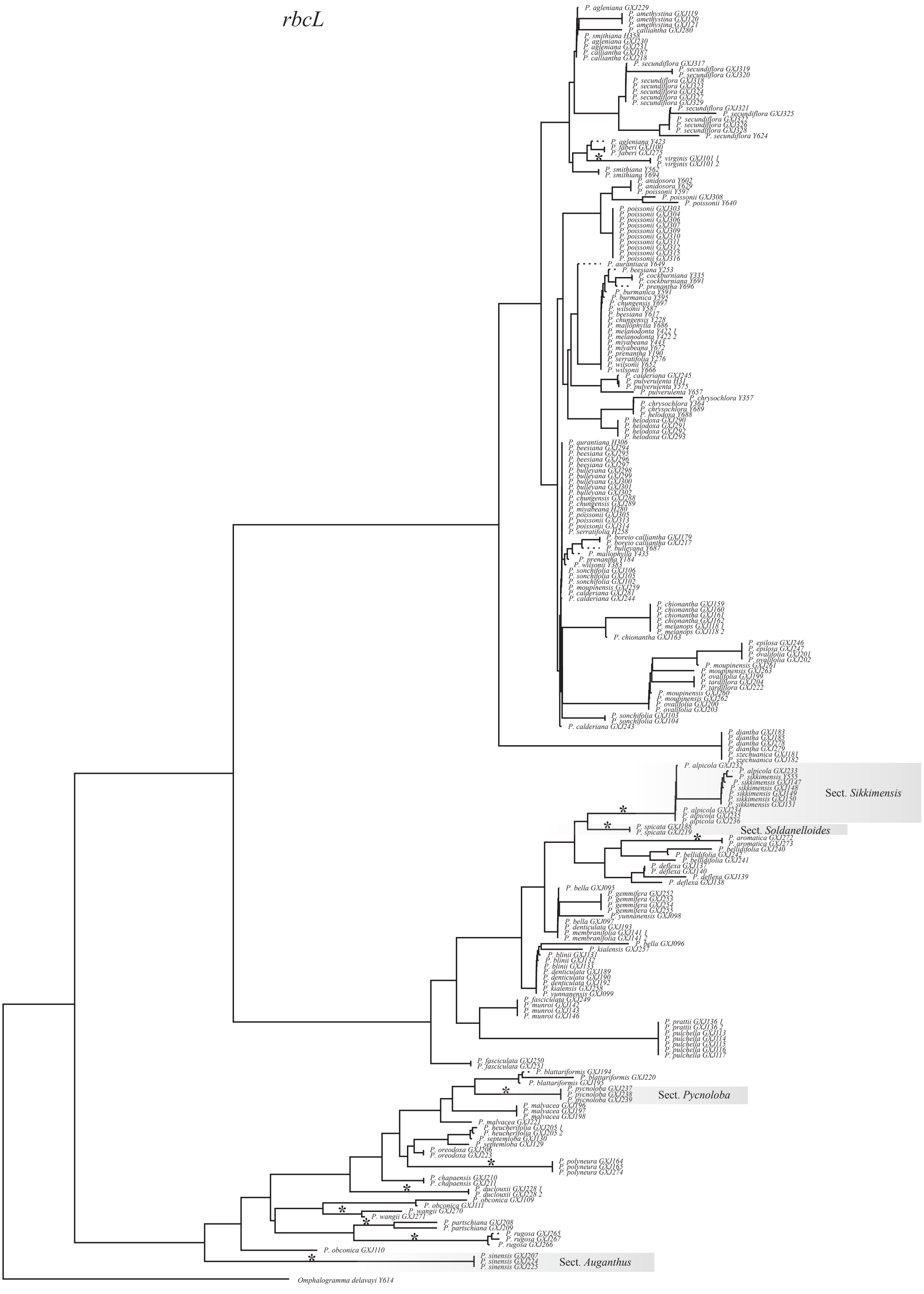
rbcL
P. agleniana GXJ229
P. amethystina GXJ119
P. amethystina GXJ120
P. amethystina GXJ121
P. calliantha GXJ280
P. smithiana H358
P. agleniana GXJ230
P. agleniana GXJ231
P. calliantha GXJ187
P. calliantha GXJ218
P. secundiflora GXJ317
P. secundiflora GXJ319
P. secundiflora GXJ320
P. secundiflora GXJ318
P. secundiflora GXJ323
P. secundiflora GXJ324
P. secundiflora GXJ327
P. secundiflora GXJ329
P. secundiflora GXJ321
P. secundiflora GXJ325
P. secundiflora GXJ322
P. secundiflora GXJ326
P. secundiflora GXJ328
P. secundiflora Y624
P. agleniana Y423
P. faberi GXJ100
P. faberi GXJ275
P. virginis GXJ101 1
P. virginis GXJ101 2
P. smithiana Y562
P. smithiana Y694
P. anidosora Y602
P. anidosora Y629
P. poissonii Y597
P. poissonii GXJ308
P. poissonii Y640
P. poissonii GXJ303
P. poissonii GXJ304
P. poissonii GXJ306
P. poissonii GXJ307
P. poissonii GXJ309
P. poissonii GXJ310
P. poissonii GXJ311
P. poissonii GXJ312
P. poissonii GXJ315
P. poissonii GXJ316
P. aurantiaca Y649
P. beesiana Y253
P. cockburniana Y335
P. cockburniana Y691
P. prenantha Y696
P. burmanica Y591
P. burmanica Y595
P. chungensis Y697
P. wilsonii Y587
P. beesiana Y617
P. chungensis Y228
P. mallophylla Y686
P. melanodonta Y422 1
P. melanodonta Y422 2
P. miyabeana Y443
P. miyabeana Y672
P. prenantha Y190
P. serratifolia Y276
P. wilsonii Y652
P. wilsonii Y666
P. calderiana GXJ245
P. pulverulenta H31
P. pulverulenta Y575
P. pulverulenta Y657
P. chrysochlora Y357
P. chrysochlora Y364
P. chrysochlora Y689
P. helodoxa Y688
P. helodoxa GXJ290
P. helodoxa GXJ291
P. helodoxa GXJ292
P. helodoxa GXJ293
P. aurantiana H306
P. beesiana GXJ294
P. beesiana GXJ295
P. beesiana GXJ296
P. beesiana GXJ297
P. bulleyana GXJ298
P. bulleyana GXJ299
P. bulleyana GXJ300
P. bulleyana GXJ301
P. bulleyana GXJ302
P. chungensis GXJ288
P. chungensis GXJ289
P. miyabeana H280
P. poissonii GXJ305
P. poissonii GXJ313
P. poissonii GXJ314
P. serratifolia H258
P. boreio calliantha GXJ179
P. boreio calliantha GXJ217
P. bulleyana Y687
P. mallophylla Y435
P. prenantha Y184
P. wilsonii Y383
P. sonchifolia GXJ106
P. sonchifolia GXJ105
P. sonchifolia GXJ102
P. moupinensis GXJ259
P. calderiana GXJ281
P. calderiana GXJ244
P. chionantha GXJ159
P. chionantha GXJ160
P. chionantha GXJ161
P. chionantha GXJ162
P. melanops GXJ118 1
P. melanops GXJ118 2
P. chionantha GXJ163
P. epilosa GXJ246
P. epilosa GXJ247
P. ovalifolia GXJ201
P. ovalifolia GXJ202
P. moupinensis GXJ261
P. moupinensis GXJ263
P. ovalifolia GXJ199
P. tardiflora GXJ204
P. tardiflora GXJ222
P. moupinensis GXJ260
P. moupinensis GXJ262
P. ovalifolia GXJ200
P. ovalifolia GXJ203
P. sonchifolia GXJ103
P. sonchifolia GXJ104
P. calderiana GXJ243
P. diantha GXJ183
P. diantha GXJ185
P. diantha GXJ278
P. diantha GXJ279
P. szechuanica GXJ181
P. szechuanica GXJ182
P. alpicola GXJ232
P. alpicola GXJ233
P. sikkimensis Y555
P. sikkimensis GXJ147
P. sikkimensis GXJ148
P. sikkimensis GXJ149
P. sikkimensis GXJ150
P. sikkimensis GXJ151
Sect. Sikkimensis
P. alpicola GXJ234
P. alpicola GXJ235
P. alpicola GXJ236
P. spicata GXJ188
P. spicata GXJ219
Sect. Soldanelloides
P. aromatica GXJ272
P. aromatica GXJ273
P. bellidifolia GXJ240
P. bellidifolia GXJ242
P. bellidifolia GXJ241
P. deflexa GXJ137
P. deflexa GXJ140
P. deflexa GXJ139
P. deflexa GXJ138
P. bella GXJ095
P. gemmifera GXJ252
P. gemmifera GXJ253
P. gemmifera GXJ254
P. gemmifera GXJ255
P. yunnanensis GXJ098
P. bella GXJ097
P. denticulata GXJ193
P. membranifolia GXJ141 1
P. membranifolia GXJ141 2
P. bella GXJ096
P. kialensis GXJ257
P. blinii GXJ131
P. blinii GXJ132
P. blinii GXJ133
P. denticulata GXJ189
P. denticulata GXJ190
P. denticulata GXJ192
P. kialensis GXJ258
P. yunnanensis GXJ099
P. fasciculata GXJ249
P. munroi GXJ142
P. munroi GXJ143
P. munroi GXJ146
P. prattii GXJ136 1
P. prattii GXJ136 2
P. pulchella GXJ113
P. pulchella GXJ114
P. pulchella GXJ115
P. pulchella GXJ116
P. pulchella GXJ117
P. fasciculata GXJ250
P. fasciculata GXJ251
P. blattariformis GXJ194
P. blattariformis GXJ220
P. blattariformis GXJ195
P. pycnoloba GXJ237
P. pycnoloba GXJ238
P. pycnoloba GXJ239
Sect. Pycnoloba
P. malvacea GXJ196
P. malvacea GXJ197
P. malvacea GXJ198
P. malvacea GXJ221
P. heucherifolia GXJ205 1
P. heucherifolia GXJ205 2
P. septemloba GXJ130
P. septemloba GXJ129
P. oreodoxa GXJ206
P. oreodoxa GXJ223
P. polyneura GXJ164
P. polyneura GXJ165
P. polyneura GXJ274
P. chapaensis GXJ210
P. chapaensis GXJ211
P. duclouxii GXJ228 1
P. duclouxii GXJ228 2
P. obconica GXJ109
P. obconica GXJ111
P. wangii GXJ270
P. wangii GXJ271
P. partschiana GXJ208
P. partschiana GXJ209
P. rugosa GXJ265
P. rugosa GXJ267
P. rugosa GXJ266
P. obconica GXJ110
P. sinensis GXJ207
P. sinensis GXJ224
P. sinensis GXJ225
Sect. Auganthus
Omphalogramma delavayi Y614
0.0005 substitutions/site

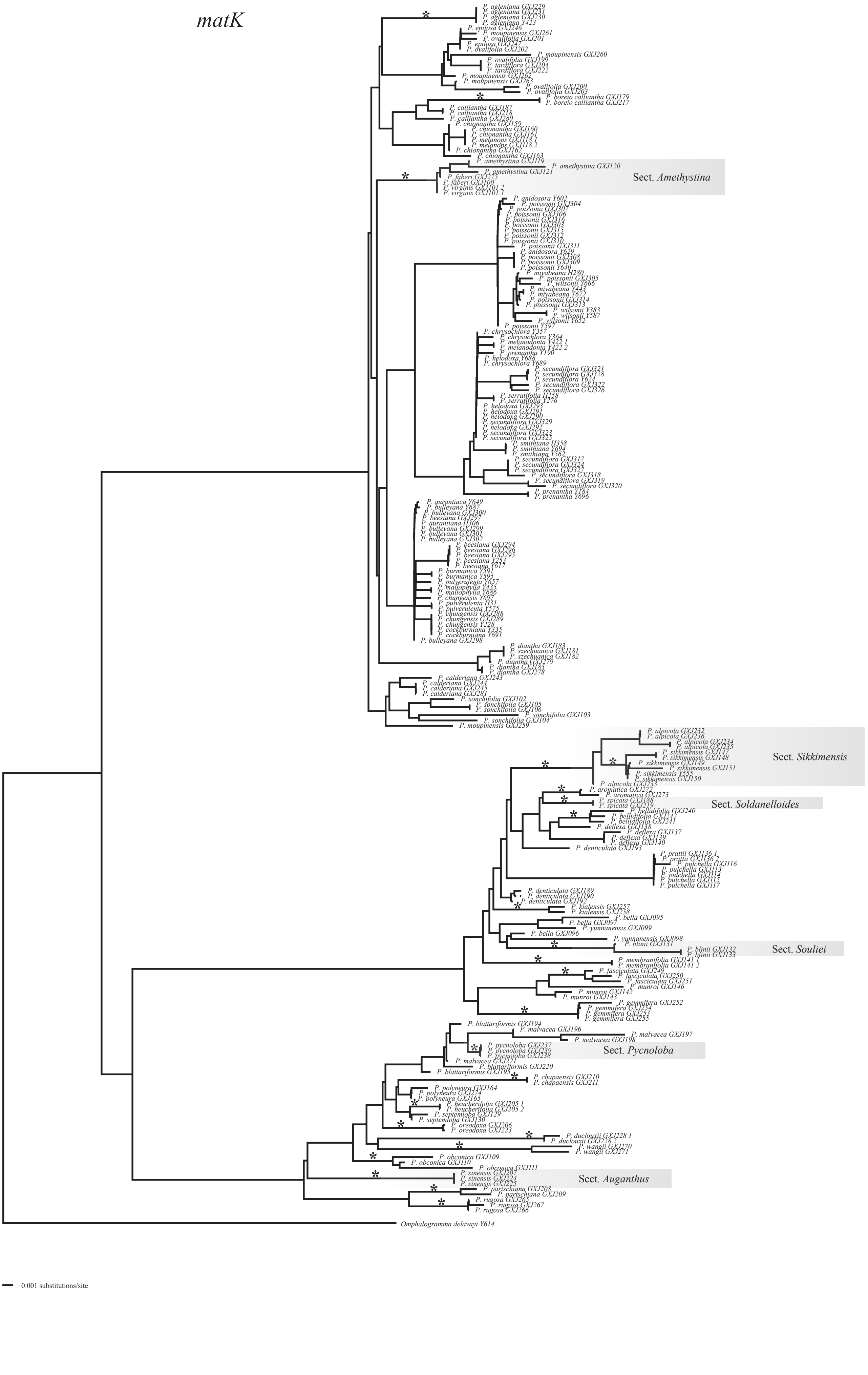

matK
Sect. Amethystina
Sect. Sikkimensis
Sect. Soldanelloides
Sect. Souliei
Sect. Pycnoloba
Sect. Auganthus
Omphalogramma delavayi Y614
0.001 substitutions/site

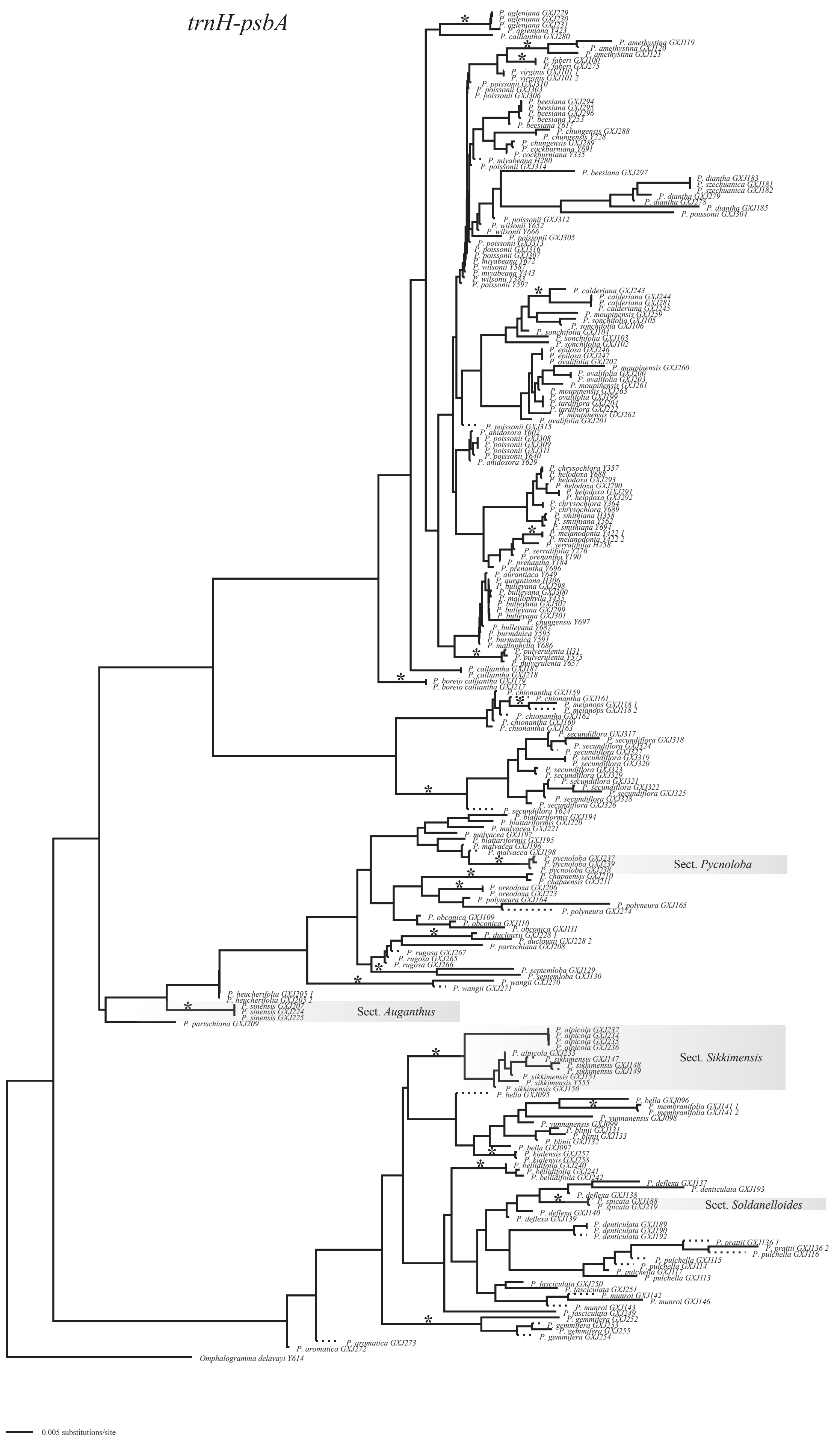
trnH-psbA
Sect. Pycnoloba
Sect. Auganthus
Sect. Sikkimensis
Sect. Soldanelloides
Omphalogramma delavayi Y614
0.005 substitutions/site

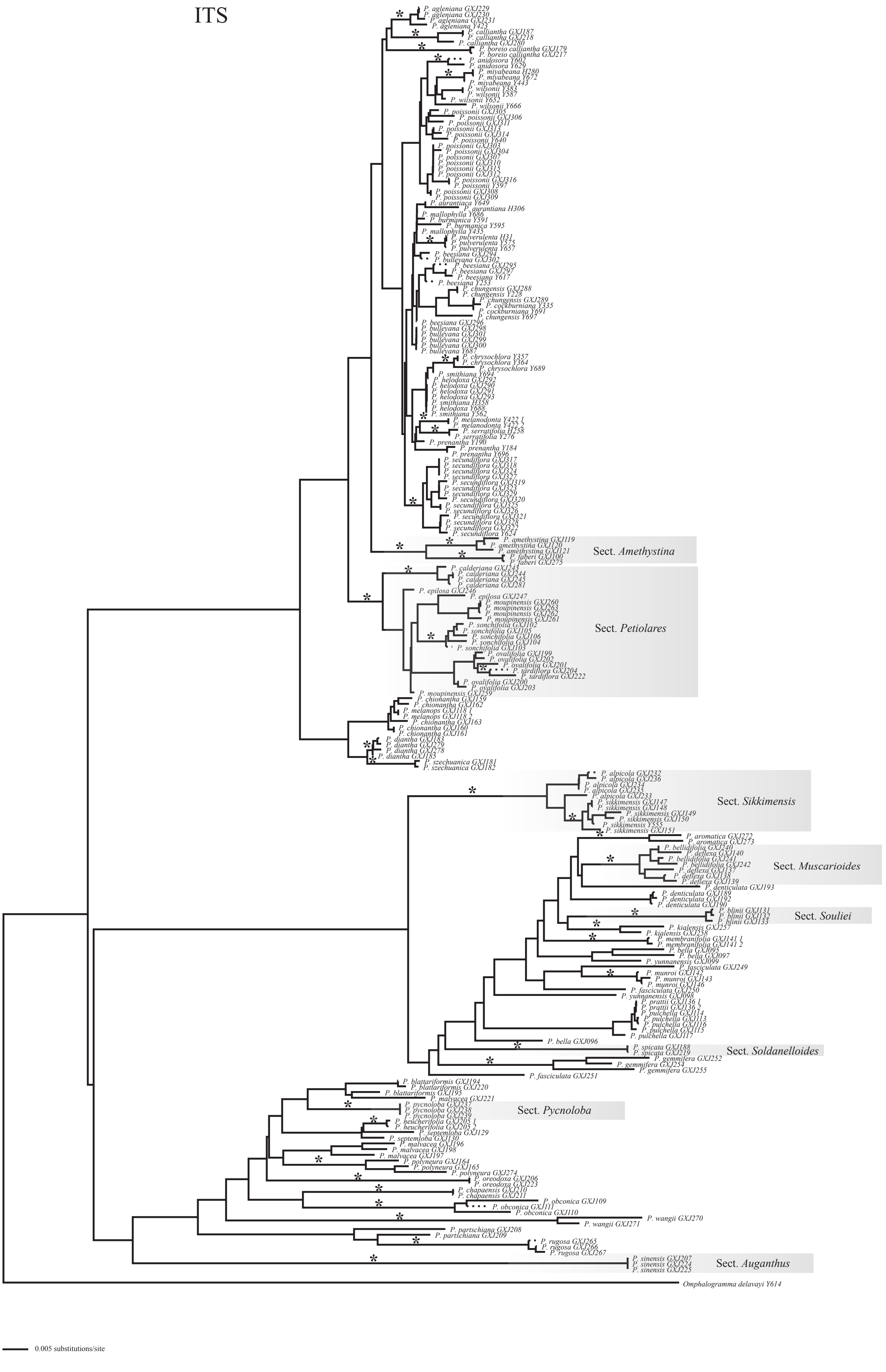
ITS
Sect. Amethystina
Sect. Petiolares
Sect. Sikkimensis
Sect. Muscarioides
Sect. Souliei
Sect. Soldanelloides
Sect. Pycnoloba
Sect. Auganthus
Omphalogramma delavayi Y614
0.005 substitutions/site

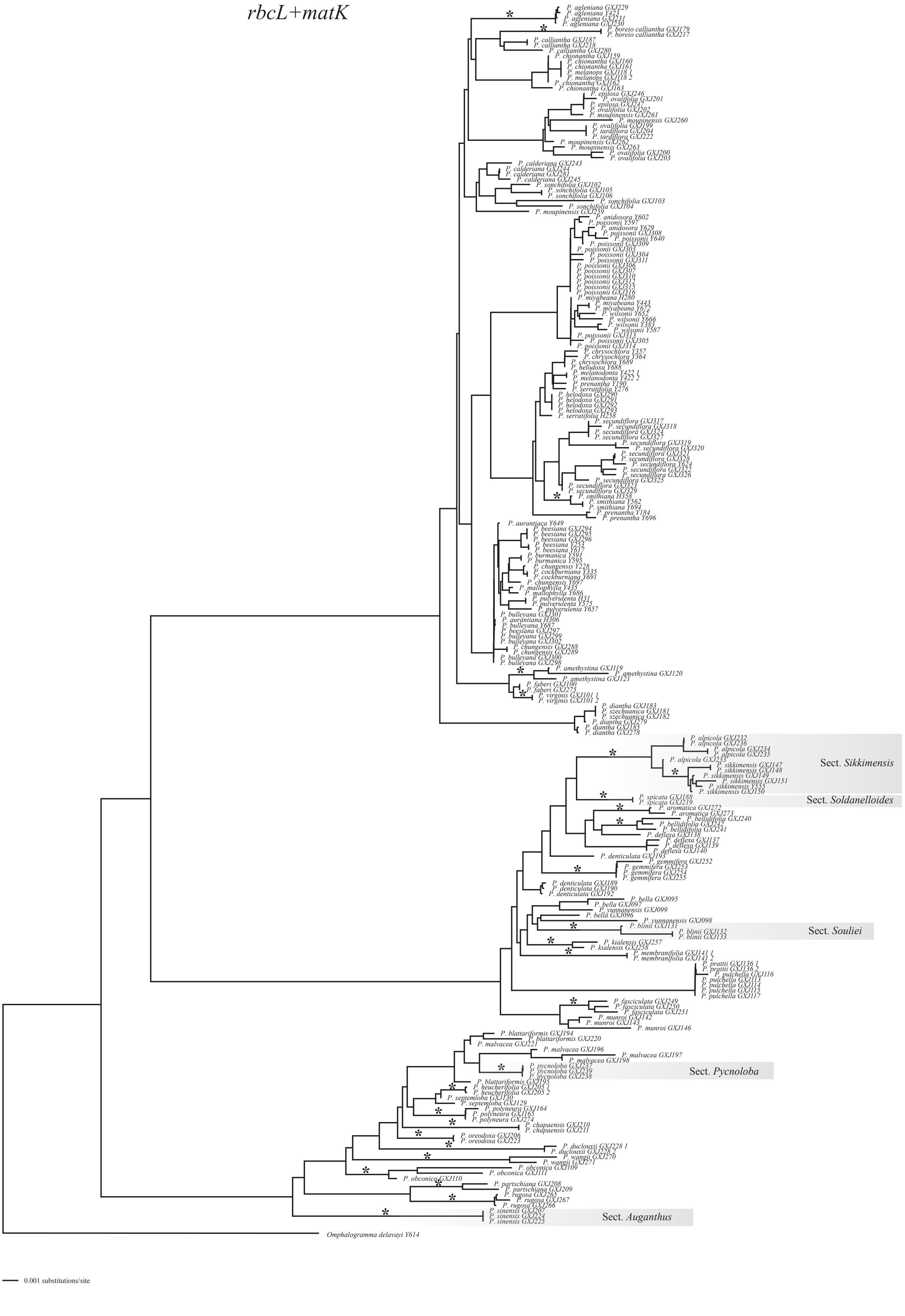

rbcL+matK
Sect. Sikkimensis
Sect. Soldanelloides
Sect. Souliei
Sect. Pycnoloba
Sect. Auganthus
Omphalogramma delavayi Y614
0.001 substitutions/site

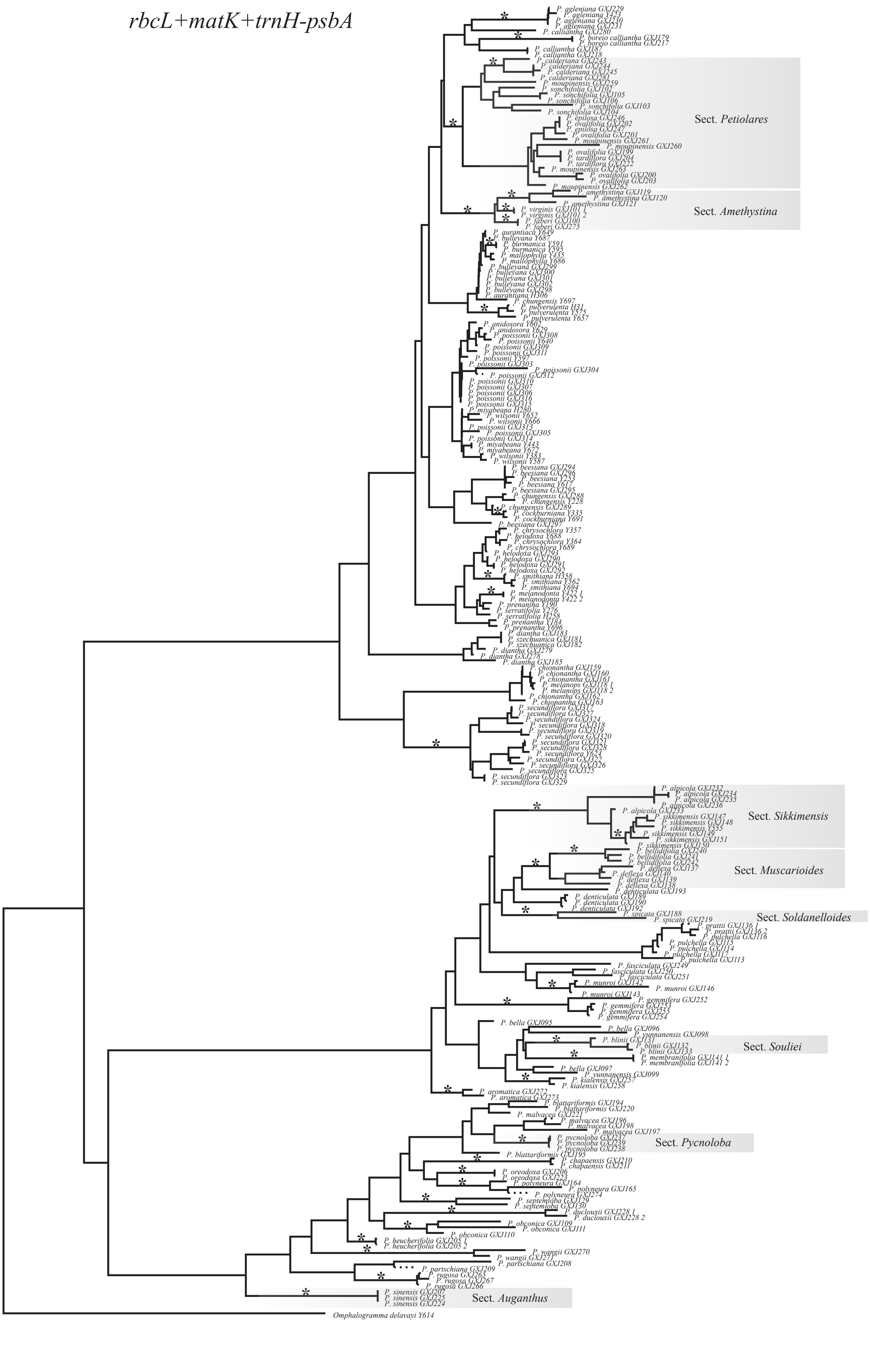

rbcL+matK+trnH-psbA
Sect. Petiolares
Sect. Amethystina
Sect. Sikkimensis
Sect. Muscarioides
Sect. Soldanelloides
Sect. Souliei
Sect. Pycnoloba
Sect. Auganthus
Omphalogramma delavayi Y614
0.001 substitutions/site

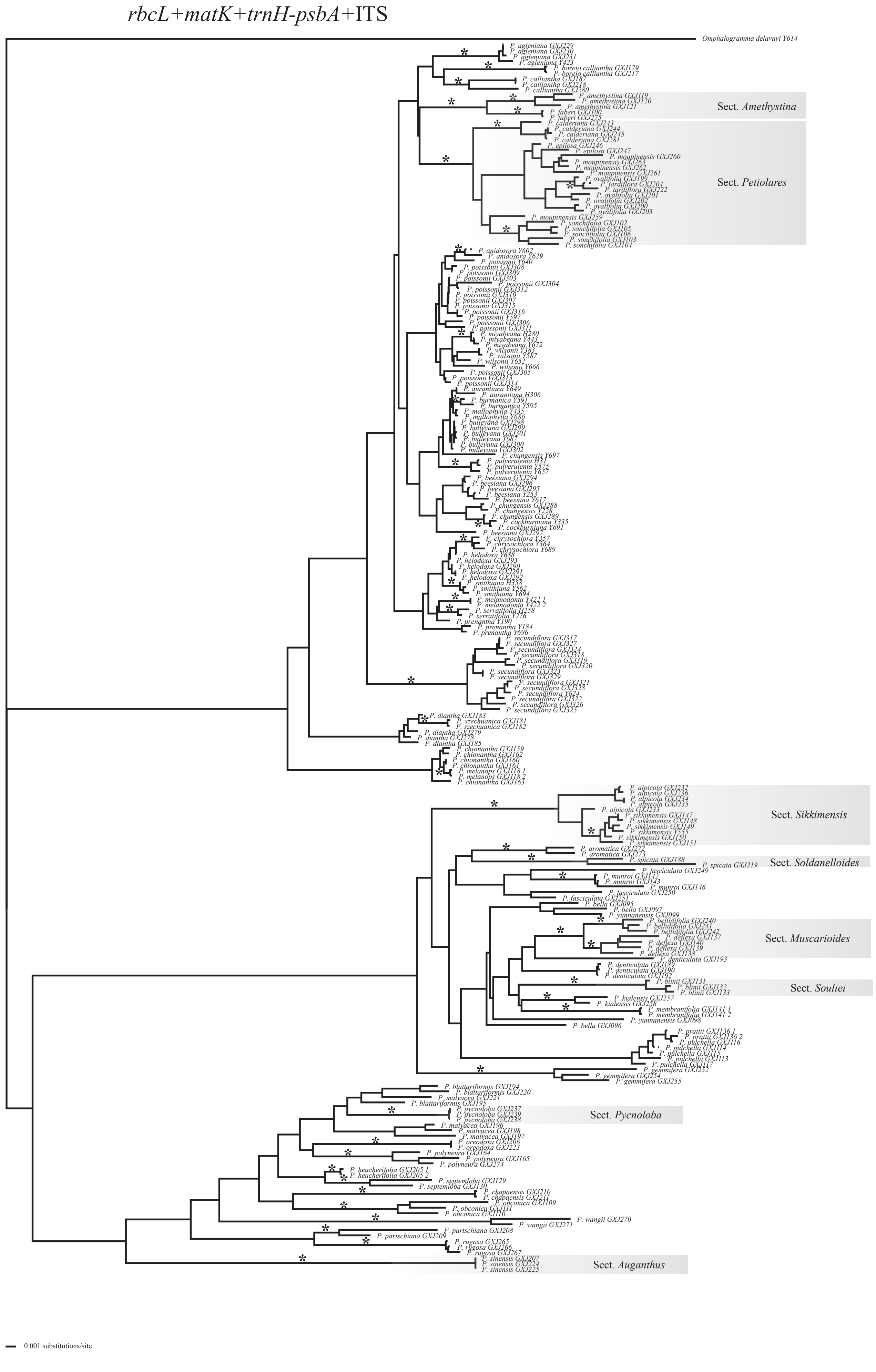

rbcL+matK+trnH-psbA+ITS
Omphalogramma delavayi Y614
Sect. Amethystina
Sect. Petiolares
Sect. Sikkimensis
Sect. Soldanelloides
Sect. Muscarioides
Sect. Souliei
Sect. Pycnoloba
Sect. Auganthus
0.001 substitutions/site
