# Supplementary material for: DNA Barcoding Evaluation and Its Taxonomic Implications in the Species-Rich Genus Primula L. in China
Source: PLoS One. 2015 Apr 13;10(4):e0122903. doi: 10.1371/journal.pone.0122903 (PMC4395239; doi:10.1371/journal.pone.0122903)
Supplement: S2 Fig — (DOCX) [file pone.0122903.s002.docx]

**Appendix S3** Three individuals of *Primula poissonii* complex and their flowers. A and D, *Primula poissonii* Y597 (voucher: Y2013016); B and E, *Primula poissonii* Y640 (voucher: Y2013083); C and F, *Primula anisodora* Y629 (voucher: Y2013062).
