# Supplementary material for: DNA Barcoding Evaluation and Its Taxonomic Implications in the Species-Rich Genus Primula L. in China
Source: PLoS One. 2015 Apr 13;10(4):e0122903. doi: 10.1371/journal.pone.0122903 (PMC4395239; doi:10.1371/journal.pone.0122903)
Supplement: S2 Table — (DOCX) [file pone.0122903.s004.docx]

**Appendix S2** Discrimination success based on different analysis methods

| Barcodes | N | Best match | | | |  | Best close match | | | | Threshold,% | PWG-Distance method | Tree-building method  (BS≧70%) |
| --- | --- | --- | --- | --- | --- | --- | --- | --- | --- | --- | --- | --- | --- |
|  |  | Correct | | Ambiguous | Incorrect |  | Correct | Ambiguous | Incorrect | No match |  |  |  |
| *rbc*L | 227 | 89 (39.20%) | 129 (56.82%) | | 9(3.96%) |  | 86 (37.88%) | 129 (56.82%) | 8 (3.52%) | 4 (1.76%) | 0.37 | 24.24% | 15.16% |
| *mat*K | 227 | 134 (59.03%) | 74 (32.59%) | | 19 (8.36%) |  | 133 (58.59%) | 74 (32.59%) | 19 (8.36%) | 1 (0.44%) | 1.11 | 42.40% | 31.82% |
| *trn*H*-psb*A | 227 | 144(63.43%) | 59 (25.99%) | | 24(10.57%) | | 144 (63.43%) | 59 (25.99%) | 22 (9.69%) | 2 (0.88%) | 2.83 | 48.40% | 42.19% |
| ITS | 222 | 182 (81.98%) | 18 (8.10%) | | 22 (9.90%) |  | 172 (77.47%) | 18 (8.10%) | 17 (7.65%) | 15 (6.75%) | 2.23 | 54.69% | 53.13% |
| ITS2 | 222 | 153 (68.91%) | 48 (21.62%) | | 21 (9.45%) |  | 144 (64.86%) | 47 (21.17%) | 16 (7.20%) | 15 (6.75%) | 2.70 | 43.75% | 39.06% |
| R + M | 227 | 164 (72.24%) | 42 (18.50%) | | 21 (9.25%) |  | 161 (70.92%) | 41 (18.06%) | 21 (9.25%) | 4 (1.76%) | 0.74 | 42.42% | 37.88% |
| R + T | 227 | 162 (71.36%) | 40 (17.62%) | | 25(11.01%) |  | 158 (69.60%) | 40 (17.62%) | 23 (10.13%) | 6 (2.64%) | 1.09 | 48.48% | 42.42% |
| R + I | 222 | 194 (87.38%) | 8 (3.60%) | | 20 (9.00%) |  | 182 (81.98%) | 8 (3.60%) | 15 (6.75%) | 17 (7.65%) | 1.21 | 57.80% | 54.69% |
| R + I2 | 222 | 171 (77.02%) | 28 (12.61%) | | 23 (10.36%) |  | 162 (72.97%) | 28 (12.61%) | 19 (8.55%) | 13 (5.85%) | 0.83 | 46.90% | 43.75% |
| M + T | 227 | 168 (74.00%) | 37 (16.29%) | | 22 (9.69%) |  | 165 (72.68%) | 37 (16.29%) | 21 (9.25%) | 4 (1.76%) | 1.37 | 50.00% | 40.90% |
| M + I | 222 | 196 (88.28%) | 9 (4.05%) | | 17 (7.65%) |  | 190 (85.58%) | 9 (4.05%) | 14 (6.30%) | 9 (4.05%) | 1.50 | 62.50% | 56.25% |
| M + I2 | 222 | 181 (81.53%) | 28 (12.61%) | | 13 (5.85%) |  | 177 (79.72%) | 28 (12.61%) | 10 (4.50%) | 7 (3.15%) | 1.32 | 50.00% | 42.19% |
| T + I | 222 | 199 (89.63%) | 2 (0.89%) | | 21(9.45%) |  | 193 (86.93%) | 1 (0.44%) | 16 (7.20%) | 12 (5.40%) | 2.00 | 60.93% | 53.13% |
| T + I2 | 222 | 177 (79.72%) | 18 (8.10%) | | 27(12.16%) | | 171 (77.02%) | 18 (8.10%) | 25 (11.26%) | 8 (3.60%) | 2.51 | 57.80% | 53.13% |
| R + M + T | 227 | 178 (78.41%) | 28 (12.33%) | | 21(9.25%) |  | 174 (76.65%) | 28 (12.33%) | 20 (8.81%) | 5 (2.20%) | 0.98 | 56.65% | 48.48% |
| R + M + I | 222 | 198 (89.18%) | 8 (3.60%) | | 16(7.20%) |  | 190 (85.58%) | 8 (3.60%) | 13 (5.85%) | 11 (4.95%) | 1.03 | 65.63% | 60.94% |
| R + M + I2 | 222 | 185 (83.33%) | 18 (8.10%) | | 19 (8.55%) |  | 180 (81.08%) | 18 (8.10%) | 15 (6.75%) | 9 (4.05%) | 0.87 | 56.25% | 50.00% |
| R + T + I | 222 | 201 (90.54%) | 1 (0.44%) | | 20 (9.00%) |  | 192 (86.48%) | 1 (0.44%) | 15 (6.75%) | 14 (6.30%) | 1.37 | 65.63% | 59.38% |
| R + T + I2 | 222 | 185 (83.33%) | 9 (4.05%) | | 28(12.61%) |  | 177 (79.72%) | 9 (4.05%) | 24 (10.81%) | 12 (5.40%) | 1.28 | 51.56% | 53.13% |
| M + T + I | 222 | 202 (90.99%) | 2 (0.89%) | | 18 (8.10%) |  | 198 (89.18%) | 2 (0.89%) | 14 (6.30%) | 8 (3.60%) | 1.46 | 59.38% | 60.94% |
| M + T + I2 | 222 | 194 (87.38%) | 10 (4.50%) | | 18 (8.10%) |  | 190 (85.58%) | 10 (4.50%) | 18 (8.10%) | 4 (1.80%) | 1.45 | 59.38% | 50.00% |
| R + M + T + I | 222 | 205 (92.34%) | 0 | | 17(7.65%) |  | 199 (89.63%) | 0 | 14 (6.30%) | 9 (4.05%) | 1.28 | 68.75% | 64.06% |
| R + M + T + I2 | 222 | 194 (87.38%) | 6 (2.70%) | | 22 (9.90%) |  | 188 (84.68%) | 6 (2.70%) | 19 (8.55%) | 9 (4.05%) | 1.04 | 62.50% | 54.69% |
